# Pollinator guilds respond contrastingly at different scales to landscape parameters of land‐use intensity

**DOI:** 10.1002/ece3.8708

**Published:** 2022-03-14

**Authors:** Kolja Bergholz, Lara‐Pauline Sittel, Michael Ristow, Florian Jeltsch, Lina Weiss

**Affiliations:** ^1^ Plant Ecology and Nature Conservation University Potsdam Potsdam Germany; ^2^ Berlin‐Brandenburg Institute of Advanced Biodiversity Research (BBIB) Berlin Germany; ^3^ Museum of Natural History Potsdam Potsdam Germany

**Keywords:** hoverflies, landscape homogenization, plant functional trait, syrphids, wild bees

## Abstract

Land‐use intensification is the main factor for the catastrophic decline of insect pollinators. However, land‐use intensification includes multiple processes that act across various scales and should affect pollinator guilds differently depending on their ecology. We aimed to reveal how two main pollinator guilds, wild bees and hoverflies, respond to different land‐use intensification measures, that is, arable field cover (AFC), landscape heterogeneity (LH), and functional flower composition of local plant communities as a measure of habitat quality. We sampled wild bees and hoverflies on 22 dry grassland sites within a highly intensified landscape (NE Germany) within three campaigns using pan traps. We estimated AFC and LH on consecutive radii (60–3000 m) around the dry grassland sites and estimated the local functional flower composition. Wild bee species richness and abundance was positively affected by LH and negatively by AFC at small scales (140–400 m). In contrast, hoverflies were positively affected by AFC and negatively by LH at larger scales (500–3000 m), where both landscape parameters were negatively correlated to each other. At small spatial scales, though, LH had a positive effect on hoverfly abundance. Functional flower diversity had no positive effect on pollinators, but conspicuous flowers seem to attract abundance of hoverflies. In conclusion, landscape parameters contrarily affect two pollinator guilds at different scales. The correlation of landscape parameters may influence the observed relationships between landscape parameters and pollinators. Hence, effects of land‐use intensification seem to be highly landscape‐specific.

## Graphical abstract

In this study, we show that different measures of land‐use intensification have contrasting, scale‐dependent effects on two important pollinator guilds, that is, wild bees and hoverflies. Therefore, our study underlines that the ecological strategy of pollinators need to be considered to mitigate the anthropogenic‐induced pollinator decline.

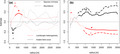

## 1 INTRODUCTION

The current decline of insect abundance and diversity alerts ecologists and the broad public worldwide (Hallmann et al., 2017; Sánchez‐Bayo & Wyckhuys, 2019; Wagner et al., 2021). In particular, the loss of pollinating insects has the potential to endanger the entire ecosystem functioning at several trophic levels across ecosystems. Approximately 87% of all wild flowering plants depend on animal pollination (Ollerton et al., 2011); therefore, insect pollinators are essential for the preservation of plant biodiversity (Biesmeijer et al., 2006; Fontaine et al., 2005) and present an extraordinarily important economic factor worldwide (Gallai et al., 2009).

The intensification of current agricultural practices is considered to be one of the main driver for the loss of pollinator biodiversity and abundances (Sánchez‐Bayo & Wyckhuys, 2019; Wagner et al., 2021). The response of pollinators to land‐use intensification should differ between pollinator guilds, since taxa highly differ in their ecological requirements and functional traits. Wild bees and hoverflies belong to the main pollinator guilds in agricultural landscapes across different habitats (Rader et al., 2020; Stanley & Stout, 2013). Wild bees are central‐place forager that search for nectar and pollen around their nests, as they need to feed their offspring (Westrich, 1996). Hereby, wild bees show a strong preference to visit flowers with specific traits, like large flower height and yellow color (Junker et al., 2013; Leong & Thorpe, 1999). Moreover, several species are oligolectic and collect pollen only from a few species (e.g., one third of wild bee species in Germany, Westrich, 1996). Hence, wild bees show specialization in their food resources to a certain extent. (Johnson & Steiner, 2000; Westrich, 1996). Hoverflies in contrast are often regarded as less specialized pollinators (e.g., Fründ et al., 2010; Jauker et al., 2019), though they also show preference toward specific flower traits (Junker et al., 2013). Hoverfly larvae feed independently on a variety of food resources and may develop across a variety of habitats, for example, aphidophagous species in arable fields or aquatic saprophagous species in eutrophic water bodies. Since adult hoverflies do not feed their offspring, they are less spatially restricted than wild bees and may therefore forage across a wide range of habitats and on much larger scales compared to wild bees, in particularly migratory species (Bankowska, 1980; Klaus et al., 2021; Lysenkov, 2009; Power et al., 2016). As a result, hoverflies are often regarded to be less susceptible to land‐use intensification than wild bees (Aguirre‐Gutiérrez et al., 2015; Blaauw & Isaacs, 2014; Jauker et al., 2009). However, solid empirical evidence is missing (e.g., Jauker et al., 2019) and a recent long‐term study reported a catastrophic decline of hoverflies during the past years in Central Europe (Hallmann et al., 2021). Despite recent attempts, our understanding of how wild bees and hoverflies are affected by different measures of land‐use intensification is limited, which hampers guidance for conservation measures and forecasting consequences of pollinator losses (Rader et al., 2020; Senapathi et al., 2017).

Land‐use intensification leads to a higher coverage of arable fields (Maskell et al., 2019). The current management regimes of these arable fields include a high frequency of mechanical disturbance, the application of pesticides and fertilizers. The resulting landscapes barely offer value for pollinating insects as food resources with the exception of short‐flowering mass events (Riedinger et al., 2014). Similarly, nesting sites are often missing. As a result, pollinators are restricted to patches of (semi‐)natural habitats within the agricultural matrix. Therefore, increasing amount of arable field coverage incorporates a reduction of food supply and habitat loss, which hampers dispersal and (re‐)colonization of habitat patches. Consequently, this leads to a decrease of pollinating insects like wild bees (Senapathi et al., 2017). In contrast, studies reported positive effects of arable field cover on hoverflies in agriculture landscapes, presumably, because larvae of some aphidophagous species may develop in agricultural fields (Brandt et al., 2017; Gabriel et al., 2010; Haenke et al., 2009). In consequence, wild bees should negatively and hoverflies positively respond to arable field cover.

Moreover, land‐use intensification may cause a loss of landscape heterogeneity (Maskell et al., 2019). The reduction of habitat diversity at the landscape scale reduces the number of potential niches and food resources; thus, landscape homogenization decreases species diversity (Fahrig et al., 2011; Senapathi et al., 2017). Although landscape heterogeneity and arable field cover may often be negatively related to each other (Tscharntke et al., 2012), high landscape heterogeneity may compensate negative effects of arable field cover (Maskell et al., 2019). Still, it remains unclear how the effect of both parameters changes with spatial scale and which is of greater importance for both pollinator guilds (but see Maskell et al., 2019). Hoverflies may suffer more from landscape homogenization, as they disperse across a wider range of habitats compared to wild bees that forage in the surrounding their nests.

Land‐use intensification may reduce the habitat quality of pollinators. Direct and indirect soil fertilization decreases overall plant species diversity (Borer et al., 2014; Maskell et al., 2010), often accompanied with a particular loss of forbs in grasslands (Maskell et al., 2010). This decline in plant diversity is also found in the context of land abandonment of unproductive habitats, such as dry grasslands, as a consequence of land‐use intensification and the (subsequent) cessation of traditional land‐use practices (Habel et al., 2013). The decline of plant diversity incorporates a reduction of possible food resources for pollinators and thus the loss of plant species may have negative consequences for pollinators (Fontaine et al., 2006). Since pollinators show preferences toward specific flower traits (see above), it is suspected that a higher variability in flower traits positively affects pollinators, rather than the taxonomic plant diversity per se (Fenster et al., 2004; Fontaine et al., 2005; Fornoff et al., 2017). Moreover, flower traits that are preferred by pollinators may attract these in the landscape thereby increasing the local abundance and richness of pollinators, for example, large flower height and yellow‐colored flowers (Donnelly et al., 1998; Leong & Thorpe, 1999; Lunau, 2014). So far, pollinator studies with a landscape context focused on flower density or plant species richness as a measures of the respective plant communities, but neglected the functional flower diversity (Grass et al., 2021), which is an essential part of how land‐use intensification affects local habitat quality for pollinators. Hereby, functional flower diversity should positively affect both pollinator guilds. Otherwise “attractive” flower traits, like high flower height and yellow coloration should have a stronger positive effect on hoverflies that migrate through the landscape.

In this study, we aim to reveal responses of two important pollinator groups to different measures of land‐use intensification, in order to get a better understanding of the underlying mechanisms of the current pollinator loss and subsequent ecosystem functioning. As a study system, we used isolated dry grassland patches that are embedded in an otherwise intensively used agricultural landscape in NE Germany. We sampled bees and hoverflies at 22 dry grassland patches within three sampling campaigns using pan traps. Further, we quantified the local flowering plant community at the time of sampling and estimated the number of flowering plants, the functional diversity of the plant flowers as well as community mean flower height and share of yellow‐colored flowers as a measure of “attractiveness.” We determined arable field cover and landscape heterogeneity on radii from 60 m to 3000 m around the traps, in order to reveal the “scale of effect” (Jackson & Fahrig, 2015), that is, the spatial scale at which the predictor has the strongest influence on the response variable. Hereby, we considered that arable field cover and landscape heterogeneity were negatively correlated to each other at larger spatial scales and discuss how this circumstance may influence the observed landscape effects on the pollinator guilds.

We hypothesize that.

1. The proportion of arable field cover has a negative effect on wild bees (species richness and abundance) and a positive effect on hoverflies,
2. Landscape heterogeneity has a stronger positive effect on hoverflies compared to wild bees,
3. The spatial scale at which arable field cover and landscape heterogeneity affect the pollinator guilds, is smaller for wild bees than for hoverflies,
4. Flower diversity positively affects wild bees in particular and flower traits that are attractive for pollinators (large flower height, yellow color) positively affect hoverflies in particular.

## 2 METHODS

### 2.1 Study area

**FIGURE 1:**
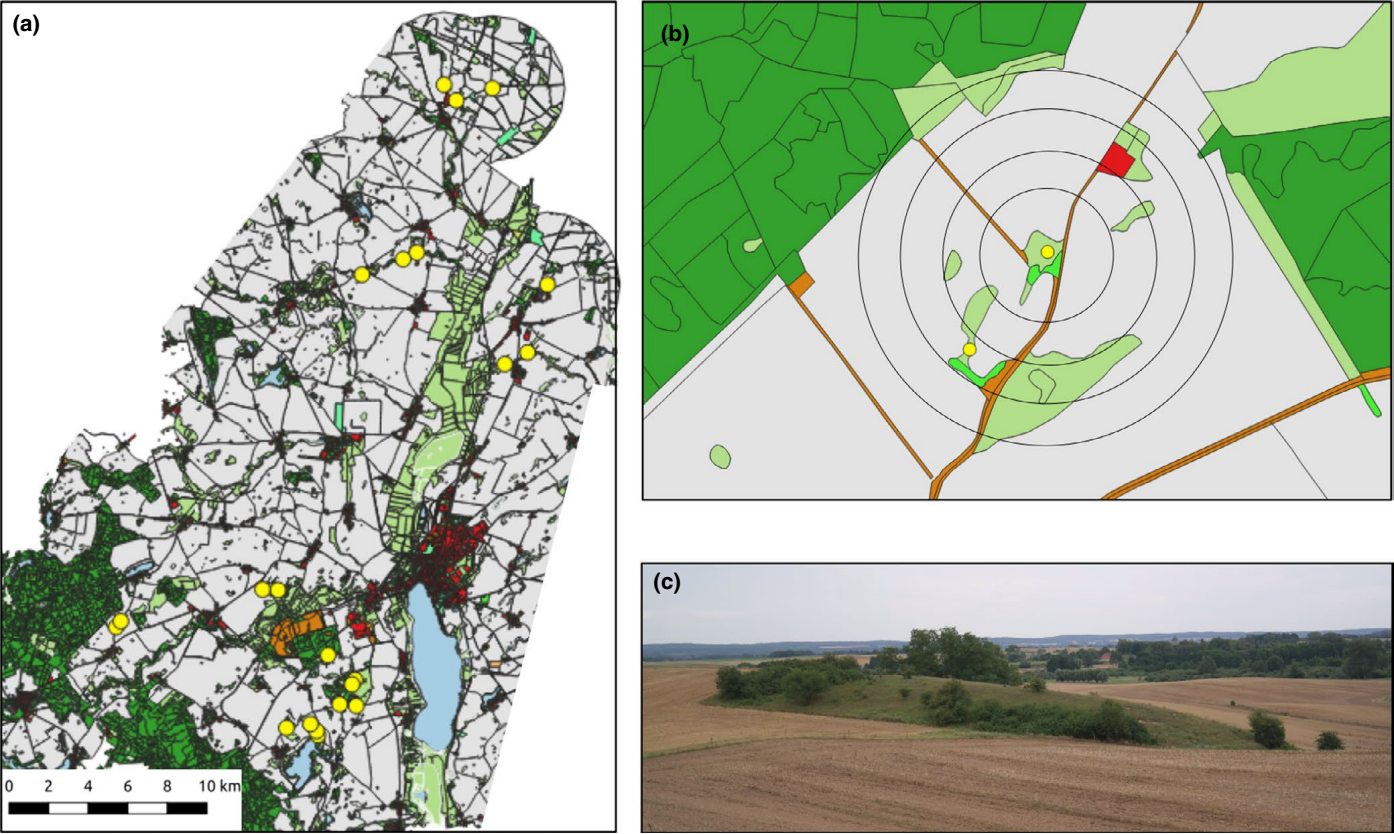
Overview of the study area and sampling design. (a) Most of the study area is used for agriculture fields (grey). Forests (dark green) make up to 13%. Grasslands (light green) are mainly intensified, wet grasslands or fallows. (b) For each sampling site, the cover of arable fields and landscape heterogeneity was calculated for different radii (60–3000 m) around the site. (c) Dry grasslands are patchy‐distributed in the landscape and are found mainly on smaller hills or slopes, often surrounded by arable fields

This study was conducted in the north‐eastern part of the federal state of Brandenburg in Germany (AgroScapeLab, http://www.zalf.de/de/struktur/eip/Seiten/AgroScapeLab.aspx, 52°52’N–53°23’N, 13°20’E–14°12’E). The study area is located at the transition zone of the west‐European oceanic and the east‐European continental climate and is characterized by a temperate climate (8.6°C) with an annual precipitation of 563 mm. The region is sparsely populated and a typical Central European agricultural landscape, to a great extent intensively used for agriculture (~ two thirds of the area, Figure 1). The dominant crop types are wheat, barley, maize, and rapeseed. The remaining area is mainly covered by forests and (mostly intensively managed) grassland. Dry grasslands are sparsely found in the region and are restricted to hills and slopes or former military areas. Overall, dry grasslands make less than one percentage of the land cover and are patchy‐distributed in the landscape as a result of the geological formation and recent management. They are remnants of the former extensive farming system of sheep grazing, and today sheep or cattle grazing and mowing is used to preserve some of the remaining patches. The sampled dry grassland plant communities belong to the class *Festuco*‐*Brometea* with some elements of the class *Koelerio*‐*Corynepheretea*, which developed under the constant land use of humans as pastures for several hundred years. Dry grassland patch sizes vary between 270 m² and 100.000 m², with a median of 5600 m².

### 2.2 Sampling design

On the basis of a pre‐survey, we selected 22 dry grassland sites that were assignable to the abovementioned plant communities. This corresponds practically to all dry grassland sites in the study region (with the exception of a current military area). At each of these sites (Figure 1), we placed three pan traps (yellow, blue, white; 19.6 × 15.4 cm with a 300 ml 8%‐Formaldehyde‐water‐dish wash‐solution) that were attached to sticks approximately 40 cm above the ground in a triangle 1 m wide triangle. The traps were installed in the center of the patches for three sampling campaigns in 2017: May (15/5–18/5/2017), June (12/6–15/6/2017) and August (15/8–18/8/2017). Each trap stayed for 48 h in the field within each sampling campaign. The weather conditions were sunny and dry throughout sampling campaigns.

Specimens were pinned and determined to species level using Amiet et al. (1996–2017) and Scheuchl (2000–2006) for wild bees and van Veen (2010) and Bot and van de Meutter (2019) for the hoverflies.

### 2.3 Flowering plant sampling

During the three sampling campaigns, we recorded all flowering forb species (henceforth plant species) nearby the pan traps, in order to characterize the local plant community. For this purpose, we placed a circle (*r* = 5 m) around the traps and recorded the presence of flowering forb species. Though our estimation of the plant community based due to time constraints on a small section of the dry grassland site, we are confident that this measure reliably estimates local plant attributes at larger scales. First, the species composition and richness showed a large variation between sites so that uncertainties due to the small scale should be negligible. Further, species richness at small scales (1–25 m²) are highly correlated to overall patch diversity in grasslands (e.g., *r* > .84, Giladi et al., 2014).

In orientation to Fornoff et al. (2017), we gathered functional flower traits of the recorded plants from Biolflor Database (Klotz et al., 2002) and Jäger (2016): UV radiation [a,b], UV reflectance [numeric 1–6], color [categorical: yellow, red, blue, white, rose, purple, violet], flowering height [continuous], and nectar access [categorical: open, half‐open, hidden] (Appendix S1).

### 2.4 Determination of landscape parameter

Both landscape parameters, arable field cover and landscape heterogeneity, were determined on the basis of the biotope mapping of the federal state of Brandenburg (https://lfu.brandenburg.de/lfu/de/aufgaben/natur/biotopschutz/biotopkartierung/). The map distinguishes a large variety of habitats. However, for this study, we used only the twelve main habitat categories: arable fields (64% cover within the study area), forests (13%), grasslands (11%), swamps (2%), built‐up areas (3%), standing waters (3%), anthropogenic immature soils (2%), deciduous copse and avenues of trees (1%), parks and cemeteries (1%), dwarf shrub heaths (<1%), streaming water including shores (<1%), and special biotopes (<1%). For small parts of our study area in the federal state of Mecklenburg‐Vorpommern, we conducted a biotope mapping by ourselves with the help of aerial images. Arable field cover is defined as the percentage of arable field cover around the traps for a specific radius. Landscape heterogeneity is defined as the Shannon diversity of main habitat types weighted by their coverage (Maskell et al., 2019). We calculated both landscape predictors for circles around the trap placements with radii from 60 m to 3000 m (each 20 m in the range from 60 – 500 m and each 50 m from 500 m – 3000 m). Arable field cover and landscape heterogeneity were shown to have a scale‐dependent correlation (Appendix S2). At small scales (<500 m), no significant (*p *< .05) correlation was found, whereas at larger scales (>500 m) both measures tended to be negatively related to each other.

### 2.5 Statistical analyses

We analyzed wild bee and hoverfly species richness and abundance, that is, number of caught individuals, in dependence on arable field cover, landscape heterogeneity and measures of the local flowering plant community. We used GLMMs with Poisson distribution and a log‐link function for all response variables (*glmer*, R‐Package lme4, Bates et al., 2020). As covariates, we included the sampling campaign as categorical fixed‐effect and study site as random intercept effect to account for the nested design of the study.

Arable field cover and landscape heterogeneity (at a specific scale) were z‐scaled and as continuous variable included in the model. We used separate models for arable field cover and landscape heterogeneity, to avoid problems with collinearity (Dormann et al., 2013), since both landscape predictors were correlated to each other at larger scales (Appendix S2). In order to reveal, how the effect of both landscape predictors changes with spatial scale (hypothesis 3), we analyzed the effect size of the respective landscape predictor at a specific spatial scale (60–3000 m) on the response variables with a series of models using the *multifit* function of Huais (2018). The model with the lowest AIC was considered to be the best model that identifies the largest effect of the landscape parameter on the response variable, that is, the scale of effect (Jackson & Fahrig, 2015). Additionally, we assessed whether the confidence interval of the landscape predictors at a specific scale crosses zero. Due to the natural patchy distribution of dry grasslands in our landscape, the circles of the landscape parameters overlap to some extent of adjacent study sites at larger scale. Hence, these landscape parameters that base on the same area cannot be considered as completely independent from each other. Therefore, in additional analyses, we assured that this form of possible pseudoreplication of the landscape parameters did not affect our findings (Appendix S3).

For the analyses of local plant attributes (hypothesis 4), we calculated four measures for each dry grassland site and sampling campaign: number of flowering species, functional diversity of flower traits (FD_trait_), community mean flower height of flowering plants, and percentage of yellow flowering species (CM_yellow_). Functional diversity was estimated with Rao's quadratic entropy (FD_trait_) of the abovementioned traits (see above, Fornoff et al., 2017). FD_trait_ showed multiple correlations with functional diversity indices obtained for single traits, but only moderate with number of flowering species (*r* = .5, see Appendix S4). The four local plant measures were z‐transformed and included in the model. For the analyses of wild bee abundance, we used a negative‐binomial error distribution to cope with one particular outlier (Appendix S4). None of the local plant community attributes were correlated to the landscape predictors at any scale.

*Apis mellifera*, the European honey bee, was excluded from all analyses. We assured that model assumptions (normality and over‐/underdispersion of residuals, heteroscedasticity, spatial autocorrelation of response variables and model residuals and zero‐inflation) were not violated with R‐package DHARMa (Hartig, 2020). All analyses were carried out in R version 4.1.0 (R Core Team, 2021).

## 3 RESULTS

We caught in total 1419 individuals of 79 wild bee species, excluding *Apis mellifera* (honey bee). Most bee individuals were caught in May (*n* = 611) followed by June (*n* = 498) and August (*n* = 310). Hoverflies were predominantly (92%) caught in August with 214 individuals of 21 species in total. The majority of the individuals (*n* = 150) belonged to aquatic‐saprophagous species (*n* = 10), whereas only 62 individuals belonged to aphidophagous species (*n* = 10). *Xylota segnis* was the only terrestrial saprophagous species with two individuals.

We observed 123 flowering plant species (Appendix S1). Flowering plant species richness ranged from one to 22 species near the pan traps with the highest number in June (mean ± *SD* = 11.55 ± 5.4) followed by August (9.05 ± 4.75) and May (7.41 ± 3.45).

**FIGURE 2:**
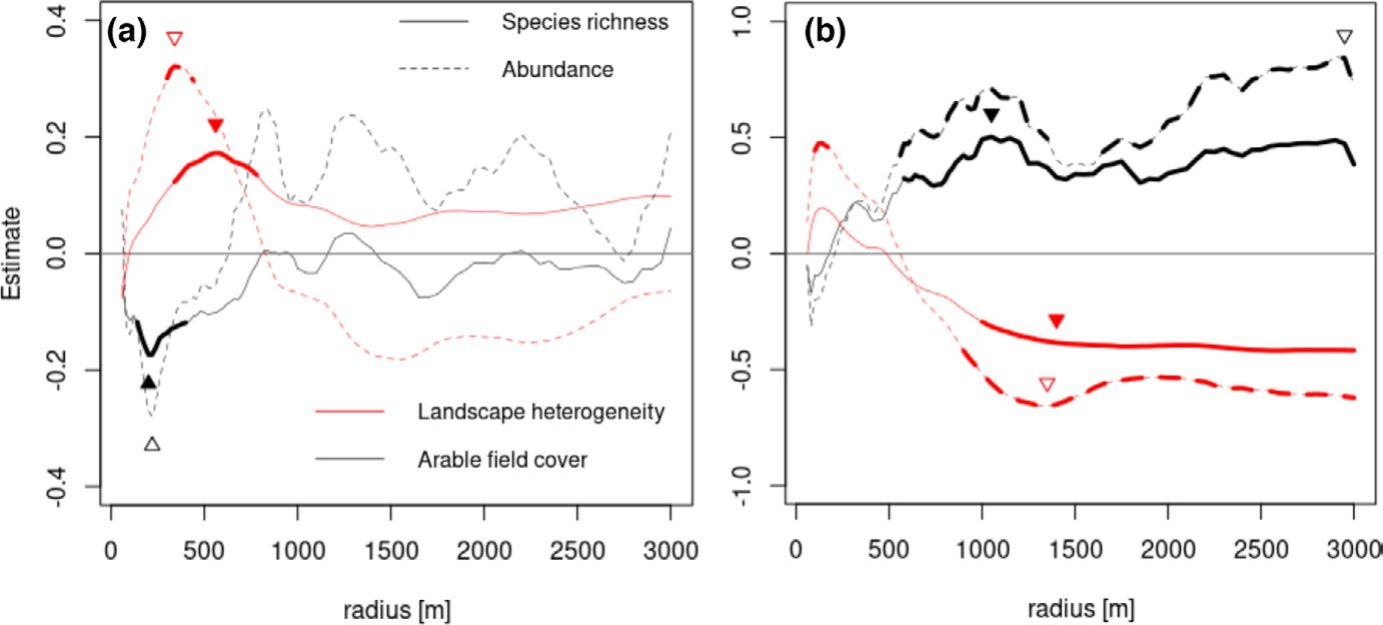
Wild bee (a) and hoverfly (b) responses to arable field cover (black) and landscape heterogeneity (red) across multiple scales (60–3000 m). The graphs show the parameter estimates of the models for both predictors on both response variables species richness (solid line) and abundance (dashed line) for each specific radius. Thick lines refer to models, in which the confidence interval of the parameter estimate does not cross zero. The triangles refer to the scale of effect, that is, the scale at which the landscape predictor has the largest effect (lowest AIC, see Figure 3). Since both arable field cover and landscape heterogeneity are negatively correlated to each other, the landscape parameters were analyzed in separate models

We found contrasting effects of arable field cover and landscape heterogeneity on both pollinator guilds (Figure 2). Arable field cover negatively affected wild bee species richness. This effect was found at small to intermediate spatial scales (140–400 m) and peaked around 200 m. Hence, dry grasslands that feature high proportion of arable fields in the surrounding show on average less wild bee species. In contrast, hoverfly species richness and abundance were positively affected by arable field cover at much larger scales (500–3000 m), supporting hypothesis 1.

**FIGURE 3:**
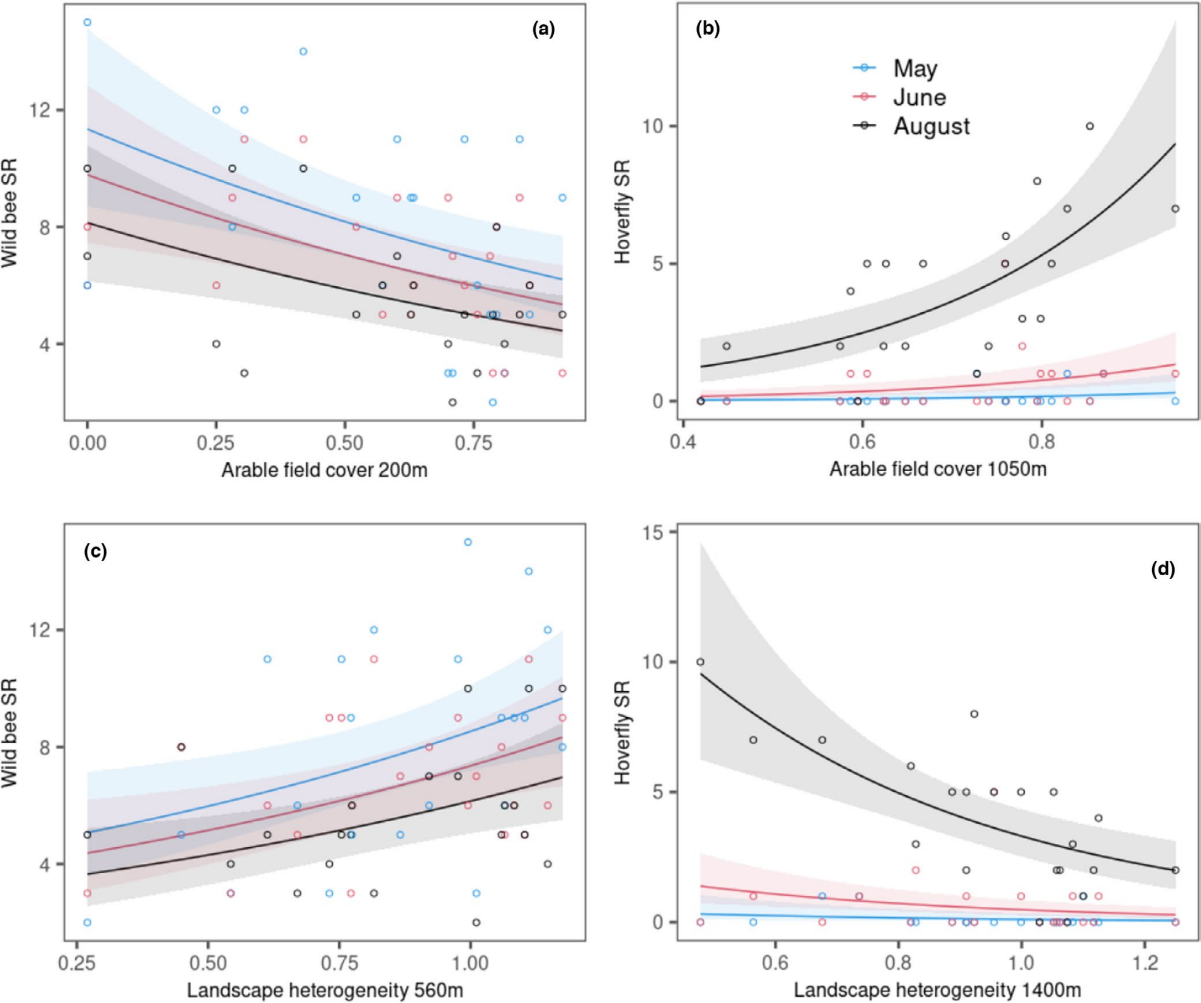
Effect of arable field cover (a, b) and landscape heterogeneity (c, d) on wild bee (a, c) and hoverfly (b, d) species richness (SR). The graphs show the relationships at the scale of effect, that is, the radius at which the landscape parameter has the largest effect on the response variable (compare Figure 1). The different colors refer to the three sampling campaigns

Landscape heterogeneity positively affected wild bees (Figures 2 and 3). Similar to the effect of arable field cover, the scale of effect for species richness had a peak at intermediate spatial scales (580 m). Hoverfly species richness and abundance were negatively affected from intermediate to large spatial scales (~500–3000 m). On these scales, arable field cover and landscape heterogeneity were negatively related to each other (Appendix S2). At small spatial scales (~120 m), landscape heterogeneity had a positive effect on hoverfly abundance, indicating that a heterogeneous environment in the vicinity of dry grasslands increase the abundance of hoverflies. Overall, we found no support that landscape heterogeneity particularly enhance hoverflies compared to wild bees (hypothesis 2). Yet, our scale‐crossing analyses showed that wild bees were mostly affected on smaller spatial scale compared to hoverflies (with the exception of the positive effect of landscape heterogeneity on abundance) supporting hypothesis 3.

**FIGURE 4:**
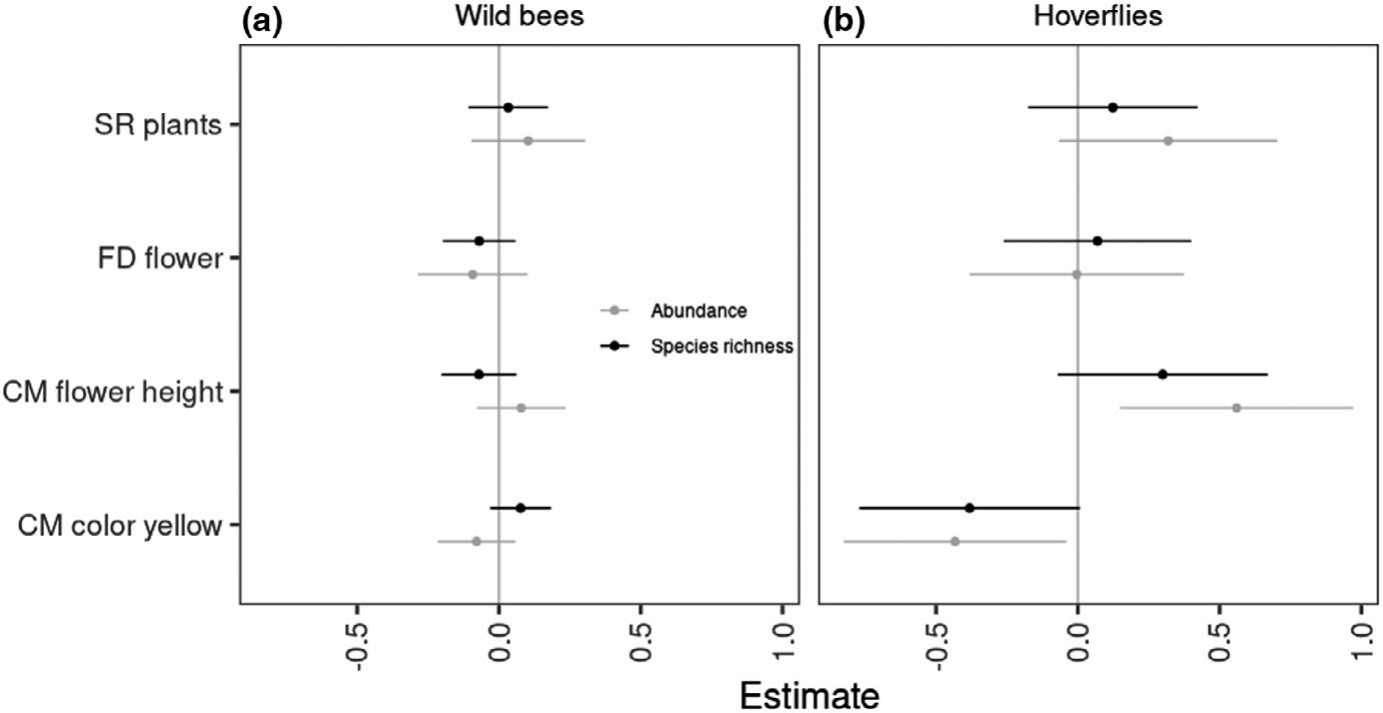
Effects of local plant community attributes on wild bees (a) and hoverflies (b). The figure shows parameter estimates and confidence intervals for species richness (black) and abundance (grey). The local plant community was recorded during pollinator samplings within a circle (*r* = 5 m) around the traps. CM, Community mean; FD, Functional diversity; SR, Species richness

Overall, we found little support that the local plant species composition and the flower attributes affected pollinator abundance and richness (hypothesis 4). Neither the number flowering plant species nor functional diversity of flower traits had a positive effect on the pollinator guilds (Figure 4). Larger flower height (high CM_flower height_) had a positive effect on hoverfly abundance, indicating that more conspicuous plant communities may attract hoverflies. In contrast to our hypothesis, a high share of yellow flowers (CM_yellow_) negatively affected hoverfly abundance.

## 4 DISCUSSION

The current decline of pollinators and other insects (Hallmann et al., 2017, 2021) calls for a thorough understanding of the underlying mechanisms to provide measures for nature conservation and mitigate losses of ecosystem services. In this study, we showed that two important pollinator guilds of Central Europe responded differently to parameters of land‐use intensification and at different spatial scales. Further, we found no support that functional diversity of local flower traits as a measure for habitat quality has positive effects on pollinators of the dry grassland patches, highlighting the role of landscape processes to maintain pollinator diversity.

### 4.1 Arable field cover

Arable field cover had a negative effect on wild bee species richness and abundance supporting previous studies that show negative effects of land‐use intensification on local pollinator diversity in agricultural landscapes (Senapathi et al., 2017). Hoverflies were, in contrast to wild bees, positively affected by the cover of arable fields, indicating that dry grasslands surrounded by a high share of arable fields, have a higher hoverfly species richness and abundance. Similar observations were made for agricultural fields and flower strips (Brandt et al., 2017; Gabriel et al., 2010; Haenke et al., 2009). These studies explain their findings mainly with the dominance of aphidophagous species whose larvae may develop in arable fields (e.g., Gabriel et al., 2010). However, in our study, the large majority of the individuals belong to aquatic‐saprophagous rather than aphidophagous species. Since hoverflies are highly mobile, they may be attracted by specific habitats, if the landscape offers no food resources (Haenke et al., 2009). As a result, hoverflies may accumulate on dry grasslands with a high proportion of arable field cover. Under this consideration, the observed “positive” effect of arable field cover on hoverfly diversity indicates simply a limitation (and concentration) of food resources within the whole landscape (Haenke et al., 2009). Wild bees as central‐place foragers are restricted to the area around the dry grassland patch of their nesting place. Hence, such concentration effects, like for hoverflies, seem to be unlikely and the negative consequences of high arable field cover, like isolation of populations, limiting food resources and pesticides, prevail. In summary, our study demonstrates that arable field cover is an important predictor for pollinators that affect both guilds contrastingly not only in agricultural ecosystems (e.g., Brandt et al., 2017) but also in (semi‐)natural habitats that present ‘biodiversity hotspots’ in Central Europe (Habel et al., 2013).

### 4.2 Landscape heterogeneity

Landscape heterogeneity should have a positive effect on pollinators, as heterogeneous landscapes provide more niches with a higher diversity of food resources and nesting sites (Dainese et al., 2019; Fahrig et al., 2011; Hopfenmüller et al., 2014; Marja et al., 2022). We predicted that hoverflies benefit more from landscape heterogeneity compared to wild bees, as they forage across a wider range of habitats (H2). In our study, wild bees were positively affected by landscape heterogeneity at intermediate spatial scales (340–780 m). Similar, hoverfly abundance was positively affect at small scales (100–140 m). However, at large scales (<750 m), landscape heterogeneity had continuously a negative effect on hoverflies. We expect that the observed negative relationship of hoverflies to landscape heterogeneity is primarily driven by hoverfly responses to a limitation of other resources in the landscape (see above), since both landscape heterogeneity and arable field cover are negatively related to each other at large scales. Hence, positive effects of landscape heterogeneity may only be important for hoverflies, if landscape heterogeneity is uncorrelated to arable field cover (as in our study for small spatial scales). In conclusion, we found no support for the hypothesis 2, which may be reasoned by specifics of our landscape, though negative correlations between arable field cover and landscape heterogeneity should be present in many areas worldwide (Tscharntke et al., 2012).

### 4.3 Scale dependency

We predicted that wild bees are affected on smaller scales compared to hoverflies (H3). Landscape heterogeneity and arable field cover affected wild bees at spatial scales that correspond to maximal foraging distances from the nest of small bees (140–350 m, Wright et al., 2015). In contrast, hoverflies that “migrate” through the landscape were affected at much larger spatial scales (>750 m, with the exception of the positive effect of landscape heterogeneity on hoverfly abundance, see below). Taking together, these results support our hypothesis and indicate that the scale of effect, that is, at which spatial scale has a landscape parameter the largest effect on a response variable, depends on the foraging behavior of pollinators. However, we detected other scales of effect than previous studies. For example, Meyer et al. (2009) found the strongest (positive) effect of landscape heterogeneity on hoverfly richness in calcareous grasslands on 250 m, while Földesi et al. (2016) observed that landscape heterogeneity positively affected hoverfly species richness at smaller spatial scales (300 m) compared to wild bees (500 m). These deviations demonstrate that the scale of effect may be primarily driven by the landscape context rather than the ecological traits of the species (Galán‐Acedo et al., 2018), preventing to deduce the scale of effect for other landscapes. Moreover, our study shows that even the direction of landscape effects may change with spatial scale. As outlined above, we assume that the negative effect of landscape heterogeneity on hoverfly abundance arises due to a negative correlation with arable field cover. Therefore, it seems likely that the scale of effect and even the direction of landscape parameter effects is driven by correlations between landscape parameters that are associated with different processes and change with scale. Therefore, we see a strong need to thoroughly analyze and report correlations of possible confounding landscape predictor across scales, in order to better understand the underlying mechanisms of how the scale of effect and direction of landscape effects arise.

### 4.4 Functional flower traits

The outstanding diversity of morphological and coloration traits in animal‐pollinated flowers is one of the most recognized examples for niche differentiation in animal communities in ecology. Hence, functional diversity of flowers is considered to positively affect pollinator diversity and *vice versa* (e.g., Blüthgen et al., 2011; Fontaine et al., 2005; Fornoff et al., 2017; Junker et al., 2013). However, we found no evidence that neither functional flower diversity nor species richness of flowering plants positively affected both pollinator guilds. This is indeed surprising, since we investigated a strong gradient from one to 23 flowering plants per study site. Similarly, to our study, Fornoff et al. (2017) neither found strong positive effects of functional flower diversity on pollinator species richness in experimental plant communities of the size 1 m². These plant communities were set even in the same landscape context and therefore local effects should appear more clearly, compared to our study. Although we are confident that our small‐scale measures (radius 5 m) reliably estimate plant functional diversity also at larger scales (see Methods), the functional diversity of a patch may play a minor role for pollinator diversity, since resources of adjacent habitats are not considered. Landscape‐wide assessments of functional flower diversity may be worth to investigate, though it may take a huge effort. In our study, the positive effect of landscape heterogeneity on both pollinator guilds may be an indication that the diversity of habitats provide different food sources (see above). Alternatively, diversity of flower traits is negligible in our system, as species are less specialized on particular flower traits than expected. Only six out of the 80 caught wild bee species are listed as oligolectic in our data set (Westrich, 2019). While a previous study within our study area observed a much higher share of oligolectic species (Saure & Berger, 2006: 28 out of 161), our pollinator community could be less specialized as a consequence of possible fragmentation and land‐use intensification (Jauker et al., 2019). Under such circumstances, the quantity of few plant species with high floral rewards rather than the diversity of flowers may maintain pollinator diversity (Bergamo et al., 2020), as indicated by the positive effect of larger flower height on hoverfly abundance, since large plants produce more flowers and are more attractive for pollinators (Donnelly et al., 1998).

### 4.5 Landscape context and pollinator composition

The specific landscape context may modulate the observed responses of pollinators. Although we are confident that similar findings (and correlations between landscape predictors) can be expected in other regions, we would like to highlight that our study area belongs to the most intensified landscapes in Europe (for comparison: 39% of the total land area of the EU is cropland, around our study patches mean of 60%) with a long history of intensive fertilization and pesticides input. Therefore, we assume that the past land‐use intensification already had a tremendous effect on the species pool in the area and the species composition in our study is only a small subset of the species pool of some decades ago. The comparison with previous studies in our study area (Hahn, 2002; Saure & Berger, 2006) indicates that not only oligolectic bee species are less represented (see above) but also common hoverflies. We observed remarkably low ratios between hoverflies and wild bee individuals compared to studies of similar study systems (our study 1419 wild bees vs. 214 hoverflies, ratio_May_ = 0.007, ratio_June_ = 0.03, ratio_August_ = 0.63; Mudri‐Stojnić et al., 2012: ratio 0.83, Jauker et al., 2009: ratio: 0.82, Jauker et al., 2019: ratio 0.95). In particular, generalist aphidophagous species (e.g., *Eupeodes corollae*, *Sphaerophoria scripta*) that occur in high densities (e.g., Hahn, 2002 for our landscape, Bankowska, 1980), are underrepresented in our study, which concurs with the decline of common hoverfly species (Hallmann et al., 2021). These deviations are most likely a consequence of the high land‐use intensity in our study region, which is the main factor for the current insect decline (Wagner et al., 2021) and question the hypothesis that hoverflies are less vulnerable than wild bees (see Jauker et al., 2019).

## 5 CONCLUSION

In conclusion, we observed contrasting and scale‐dependent responses of wild bees and hoverflies to measures of land‐use intensification, with no particular effects of local flower diversity of plants. As a consequence, pollination service on dry grasslands should change with the surrounding landscape. In homogeneous landscapes with a high share of arable field cover, insect pollination should occur less frequent, due to lower numbers of wild bee individuals and species. Although hoverflies may concentrate particularly in these landscapes at dry grasslands they cannot compensate absence of wild bees, as they appear only in late summer in reasonable amounts and have different flower preferences (Brandt et al., 2017; Junker et al., 2013). Moreover, the comparison with historical data indicate that oligolectic wild bee species and generalist hoverflies may be declining, presumably due to high land‐use intensification. In order to achieve a better understanding of how land‐use intensification affects pollinators, we advocate to (a) acknowledge that landscape effects may differ between landscapes and (b) analyze therefore possibly confounding landscape parameters across scales.

## CONFLICT OF INTEREST

The authors declare no conflict of interest.

## AUTHOR CONTRIBUTION

**Kolja Bergholz:** Conceptualization (lead); Data curation (equal); Formal analysis (lead); Investigation (lead); Methodology (lead); Project administration (equal); Supervision (lead); Visualization (lead); Writing – original draft (lead). **Lara‐Pauline Sittel:** Data curation (equal); Formal analysis (supporting); Investigation (equal); Methodology (supporting); Visualization (supporting); Writing – original draft (supporting). **Michael Ristow:** Data curation (equal); Investigation (equal); Supervision (supporting); Writing – original draft (supporting). **Florian Jeltsch:** Conceptualization (supporting); Funding acquisition (lead); Investigation (supporting); Methodology (supporting); Project administration (equal); Supervision (supporting); Writing – original draft (supporting). **Lina Weiss:** Conceptualization (supporting); Data curation (supporting); Investigation (supporting); Methodology (supporting); Project administration (equal); Supervision (supporting); Validation (supporting); Writing – original draft (supporting).

## Supporting information

Supplementary Material

## Data Availability

We stored all collected data at the open research database of ZALF (https://doi.org/10.4228/zalf.6x5w‐yc93).
