# Supplementary material for: Pollinator guilds respond contrastingly at different scales to landscape parameters of land‐use intensity
Source: Ecol Evol. 2022 Mar 14;12(3):e8708. doi: 10.1002/ece3.8708 (PMC8928882; doi:10.1002/ece3.8708)

Supplementary information: Bergholz et al. - Pollinator guilds respond contrastingly at different scales to landscape parameters of land-use intensity

Appendix 1

Tab. S1 List of animal-pollinated forb species that flowered during the three sampling campaigns including their functional flower traits taken from Biolflor database (Klotz et al. 2002) and Jäger (2016).

Species	Color	UV pattern	UV blossom	Flower height [cm]	Nectar access
Achillea millefolium	white	b	1	50	H
Acinos arvensis	violet	a	2	20	H
Agrimonia eupatoria	yellow	a	5	55	O
Ajuga genevensis	blue	b	2	20	H
Alyssum alyssoides	yellow	NA	NA	17	OH
Anchusa arvensis	blue	b	1	27	H
Anthemis tinctoria	yellow	b	1	45	H
Arabidopsis thaliana	white	b	1	23	OH
Arabis glabra	white	b	1	80	OH
Arabis hirsuta	white	b	2	20	OH
Arenaria serpyllifolia	white	b	2	7	OH
Armeria maritima	rose	b	2	17	H
Asperula tinctoria	white	a	3	50	H
Calluna vulgaris	violet	NA	2	65	H
Campanula persicifolia	blue	NA	5	50	H
Campanula sibirica	blue	NA	NA	27	H
Capsella bursa pastoris	white	b	2	36	OH
Centaurea jacea	blue	a	2	27	H
Centaurea scabiosa	blue	a	2	60	H
Centaurea stoebe	purple	a	2	37	H
Cerastium arvense	white	a	2	23	OH
Cerastium glomeratum	white	b	2	17	OH
Cerastium glutinosum	white	b	0	11	OH
Cerastium semidecandrum	white	b	0	11	OH
Cirsium vulgare	purple	b	1	90	H
Consolida regalis	blue	a	5	37	H
Convolvulus arvense	white	b	2	50	H
Conyza canadensis	white	b	2	65	H
Crataegus monogyna	white	b	1	150	O
Cyanus segetum	blue	a	6	60	H
Cynoglossum officinale	violet	a	3	55	H
Daucus carota ssp carota	white	b	1	65	O
Descurainia sophia	yellow	b	1	40	OH
Dianthus carthusianorum	purple	b	1	30	H
Echium vulgare	rose	b	5	50	H
Erigeron muralis	rose	b	1	35	H
Erodium cicutarium	rose	a	5	20	H
Euphrasia stricta	violet	a	2	15	H
Falcaria vulgaris	white	b	2	55	O
Filipendula vulgaris	white	b	1	45	O

Fragaria viridis	white	b	1	13	OH
Galium album	white	b	2	65	O
Galium aparine	white	b	2	100	O
Galium verum	yellow	b	1	45	O
Geranium molle	rose	NA	NA	27	H
Geranium pusillum	rose	a	4	19	H
Helianthemum nummularium	yellow	a	5	15	O
Helichrysum arenarium	yellow	b	1	20	H
Hieracium pilosella	yellow	b	1	15	H
Hypericum perforatum	yellow	a	5	45	O
Hypochaeris radicata	yellow	a	4	30	H
Jasione montana	blue	b	1	40	H
Knautia arvensis	blue	b	1	55	H
Lamium purpureum	purple	a	2	30	H
Leontodon hispidus	yellow	a	5	35	H
Leucanthemum vulgare	white	b	1	50	H
Lotus corniculatus	yellow	b	1	23	H
Medicago lupulina	yellow	b	1	30	H
Medicago minima	yellow	b	1	30	H
Medicago x varia	violet	b	1	55	H
Myosotis ramosissima	blue	b	1	15	H
Myosotis stricta	blue	b	1	13	H
Ononis repens	red	a	2	45	H
Origanum vulgare	purple	b	2	40	H
Papaver argemone	red	a	5	23	O
Papaver dubium	red	a	6	50	O
Papaver rhoeas	red	a	4	50	O
Petrorhagia prolifera	rose	a	2	35	H
Peucedanum oreoselinum	white	b	2	100	O
Picris hieracioides	yellow	a	5	55	H
Plantago lanceolata	brown	b	1	27	NA
Plantago media	white	b	2	63	O
Polygala comosa	blue	b	2	15	H
Potentilla argentea	yellow	a	4	25	H
Potentilla heptaphylla	yellow	a	4	13	H
Potentilla incana	yellow	a	4	10	H
Primula veris	yellow	b	1	20	H
Ranunculus bulbosus	yellow	b	1	30	OH
Salvia pratensis	blue	b	2	45	H
Sanguisorba minor	red	b	2	50	NA
Saxifraga granulata	white	b	2	27	OH
Saxifraga tridactylites	white	NA	NA	10	O
Scabiosa columbaria	blue	b	1	43	H
Sedum acre	yellow	a	6	9	OH
Senecio jacobaea	yellow	a	6	65	H
Senecio vernalis	yellow	a	5	60	H
Silene latifolia	white	a	3	65	H
Silene otites	yellow	NA	NA	40	H
Silene vulgaris	white	a	3	35	H
Stachys recta	white	b	2	40	H
Teesdalia nudicaulis	white	b	2	14	OH
Thymus pulegioides	purple	b	2	17	H

Tragopogon pratensis	yellow	a	3	40	H
Trifolium alpestre	red	b	1	30	H
Trifolium arvense	white	b	1	19	H
Trifolium campestre	yellow	b	2	17	H
Trifolium dubium	yellow	b	2	30	H
Trifolium montanum	white	b	1	27	H
Valerianella locusta	blue	b	2	7	H
Verbascum densiflorum	yellow	a	5	125	O
Veronica arvensis	blue	a	5	14	H
Veronica chamaedrys	blue	a	5	23	O
Veronica spicata	blue	b	2	23	H
Vicia angustifolia	purple	NA	NA	38	H
Vicia cracca	violet	b	2	75	H
Vicia hirsuta	white	b	2	38	H
Vicia lathyroides	violet	NA	NA	10	H
Vicia tenuifolia	blue	b	2	105	H
Vicia tetrasperma	rose	b	2	38	H
Viola arvensis	white	b	1	13	H

Appendix 2

Fig. S1 Pearson's correlation coefficient between arable field cover and landscape heterogeneity across different scales. Red dots indicate that both variables are significantly (p<0.05) correlated to each other.

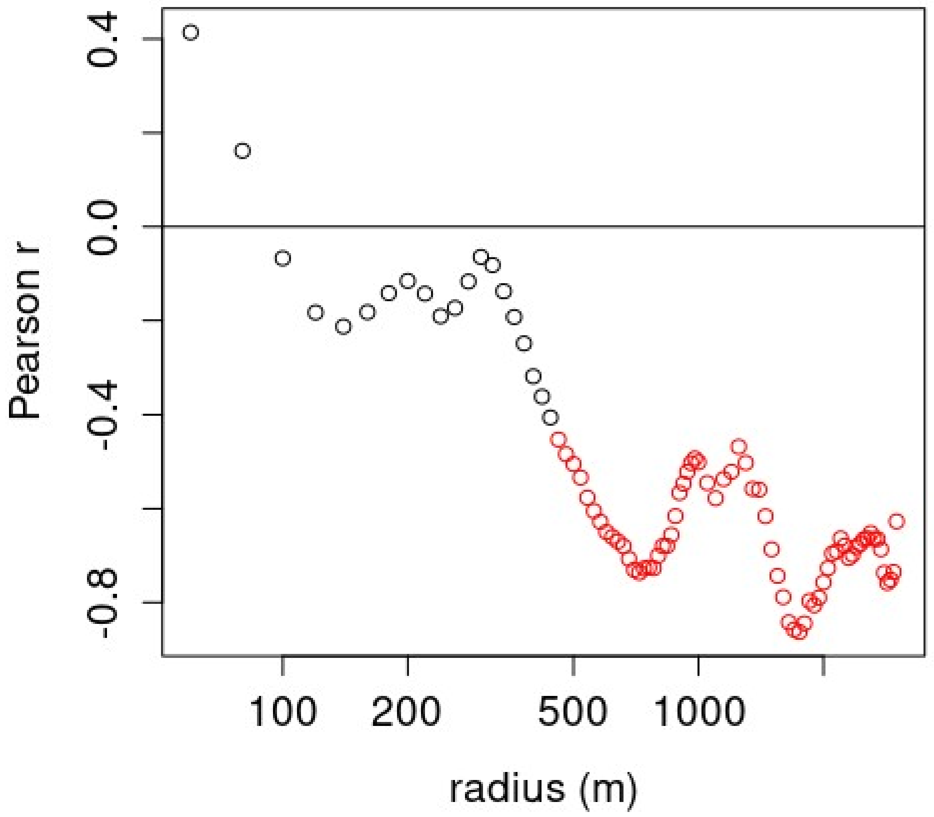

Appendix 3

Fig. S2 The dry grasslands in the region are rare and occur in clusters (see Fig. 1 in main manuscript). As a result, our sampled data is spatially clustered, though we found no evidence that our response variables or residuals of the models show spatial autocorrelation. However, landscape parameters (arable field cover and landscape heterogeneity) were determined for large spatial scales. Therefore, the circles around study sites that were used to calculate the landscape parameters overlap to some extent of adjacent study sites. As a results, landscape parameters that use the same area during the calculations cannot be considered as completely independent from each other. In order to assure that this obstacle does not influence our results, we did further analyses that incorporate the clustered sampling design. For this purpose, we determined buffers with radii of 500m and 1000m around the study sites. If the buffers of two or more study sites overlap, they were assigned to the same landscape cluster, i.e. study sites for which the landscape parameters are not completely independent from each other. We identified twelve clusters for 500m buffers and seven clusters for 1000m buffers. The same clusters were identified, if other radii were used (500m: ~250m – 700m, 1000m: 800m – 1500m). We used the same models as explained in the main manuscript, but included additionally the landscape cluster (either 500m or 1000m) as random effect, in which study site is nested within the landscape cluster. Hence, these models refer to a classical hierarchically nested block design. We compared the model predictions of the different models (manuscript model, 500m cluster and 1000m cluster, see Figure S2 below). Overall, the different models predicted similar relationships, except of hoverfly responses at large spatial scales (>1500m) were not that clear in the 1000m cluster models. However, these deviations do not affect our main conclusions that landscape parameters affect hoverflies on larger spatial scales compared to wild bees and that landscape heterogeneity is negatively and arable field cover is positively associated with hoverfly species richness and abundance. Please note that the line types deviate from the line types in the main manuscript.

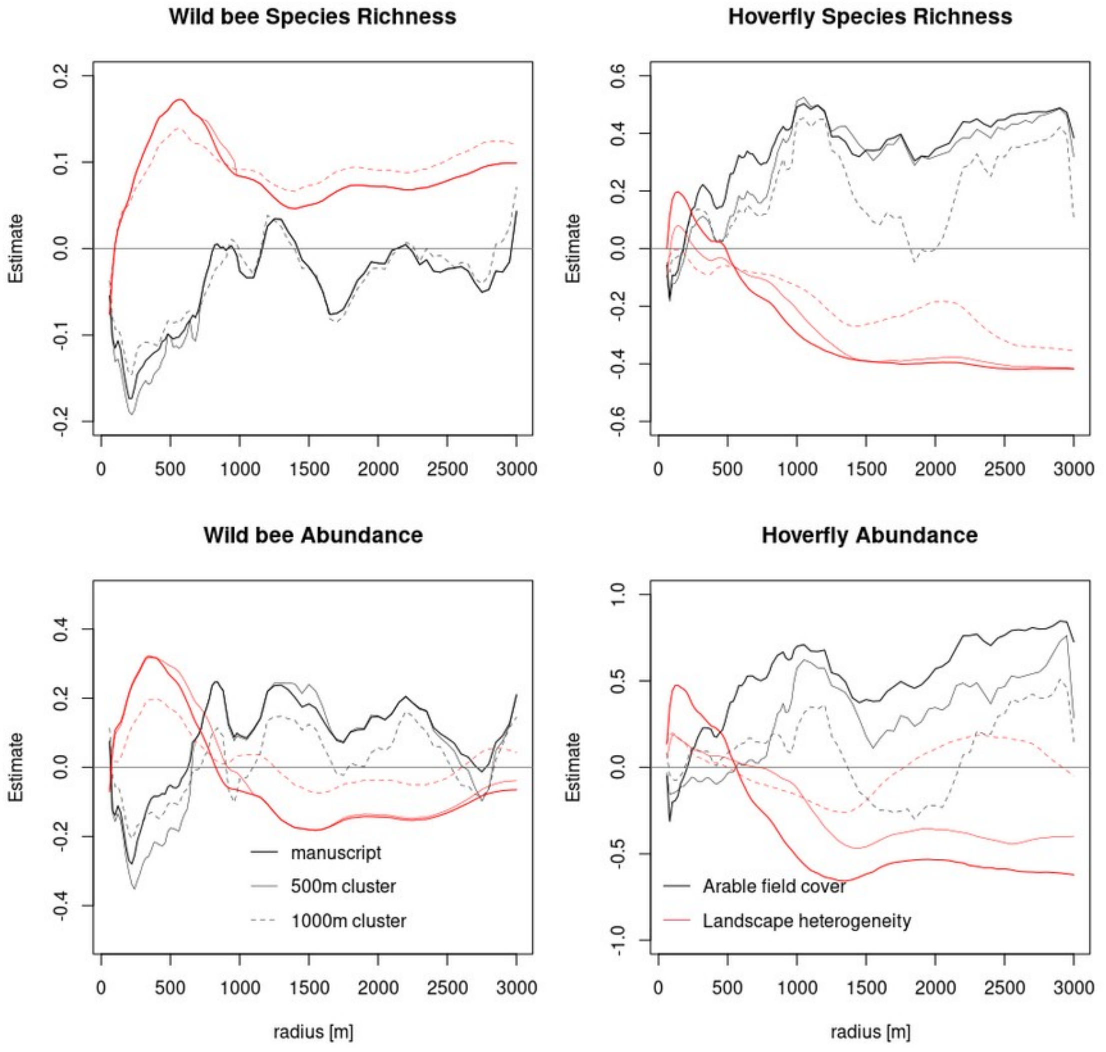

Appendix 4

Fig. S3 Correlations between different indices that quantify the functional flower composition of animal-pollinated plants (see Table S1). nbsp.fl – Number of flowering plant species, FD.all – Functional diversity that considers all listed flower traits.

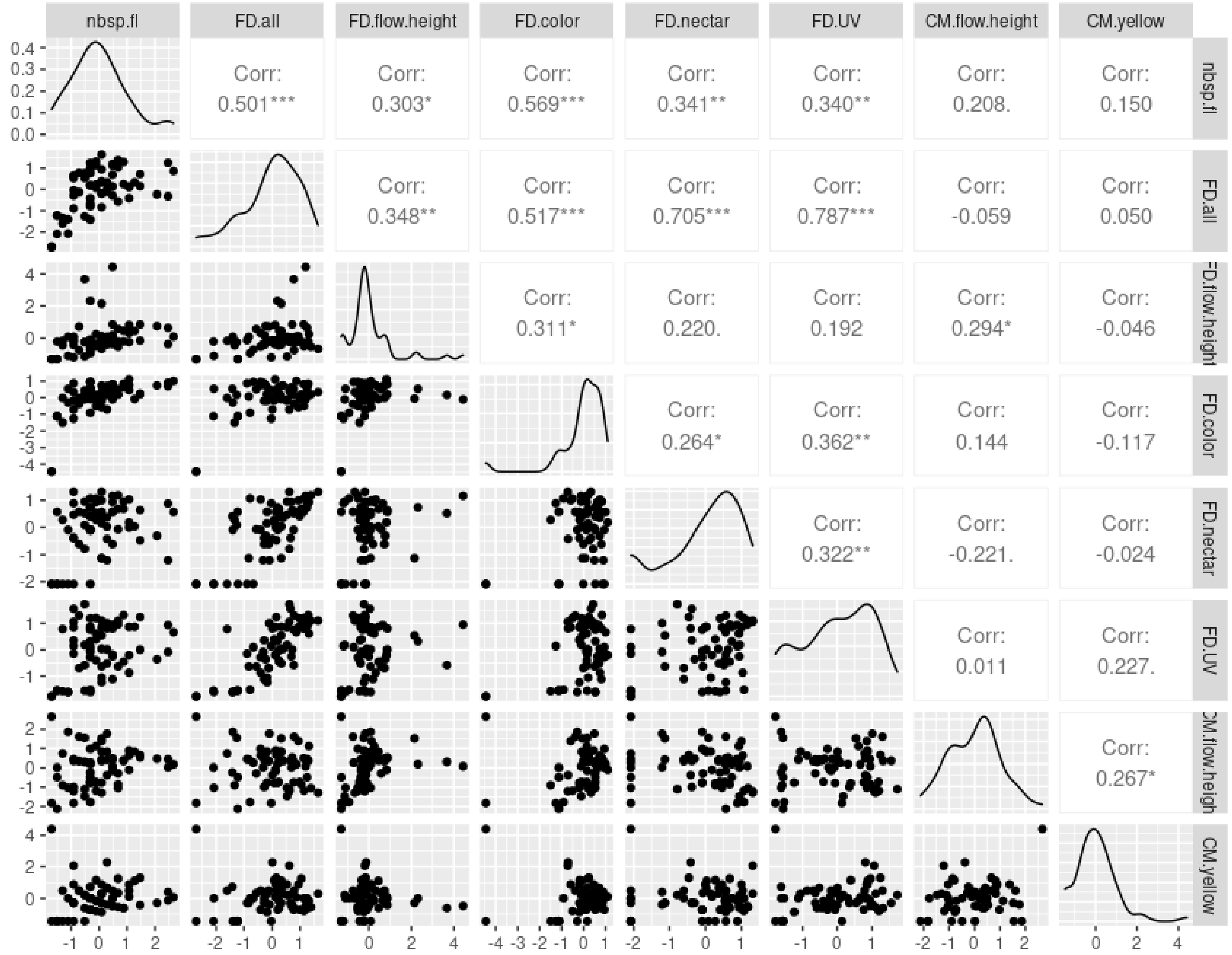

Fig. S4 Relationship between $CM_{flower\ height}$ (scaled) and wild bee abundance. The scatterplot reveals one outlier (red) within the statistical analysis that has a large effect on the results. If the outlier is included in the model with poisson-error, the model estimates a highly significant ($p<0.001$) positive effect of $CM_{flower\ height}$ on wild bee abundance (parameter estimate= 0.17, CI=[0.09; 0.24]). If a negative-binomial error distribution is taken (as reported in the main manuscript), the effect is less strong and the confidence interval crosses zero. The outlier belongs to a dry grassland patch with the lowest observed plant species richness found in this study (n=1); a mono dominance of *Senecio vernalis* at the time of sampling. Nearby the traps, a large colony of *Lasioglossum morio* was found that made more than 95 percent of the caught individuals.

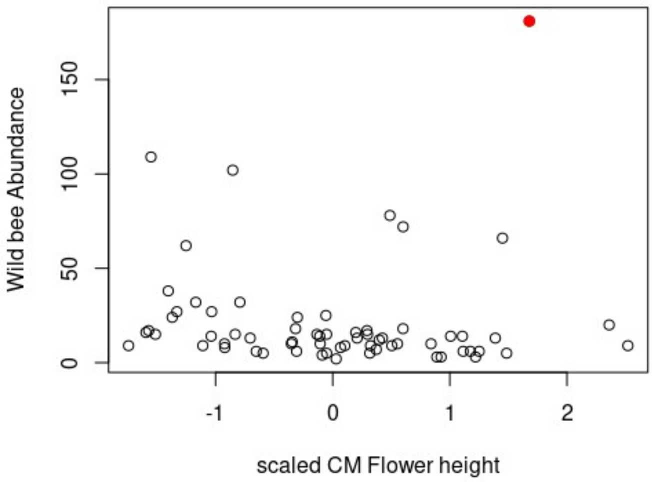

Supplement: Supplementary file 1 — Supplementary Material [file ECE3-12-e8708-s001.pdf]
